# Peripheral, Central and Behavioral Responses to the Cuticular Pheromone Bouquet in *Drosophila melanogaster* Males

**DOI:** 10.1371/journal.pone.0019770

**Published:** 2011-05-20

**Authors:** Tsuyoshi Inoshita, Jean-René Martin, Frédéric Marion-Poll, Jean-François Ferveur

**Affiliations:** 1 Centre des Sciences du Goût et de l'Alimentation, Unité Mixte de Recherche-6265 Centre National de la Recherche Scientifique, Unité Mixte de Recherche-1324 Institut National de la Recherche Agronomique, Université de Bourgogne, Dijon, France; 2 Laboratoire de Neurobiologie and Développement, Unité Propre de Recherche 3294, Centre National de la Recherche Scientifique - Institut de Neurobiologie Alfred Fessard, Gif-sur-Yvette, France; 3 Physiologie de l'Insecte: Signalisation et Communication, Unité Mixte de Recherche-1272 Institut National de la Recherche Agronomique/Université Pierre et Marie Curie, Versailles, France; 4 AgroParisTech, Département Science de la Vie et Santé, Paris, France; University of Arizona, United States of America

## Abstract

Pheromonal communication is crucial with regard to mate choice in many animals including insects. *Drosophila melanogaster* flies produce a pheromonal bouquet with many cuticular hydrocarbons some of which diverge between the sexes and differently affect male courtship behavior. Cuticular pheromones have a relatively high weight and are thought to be — mostly but not only — detected by gustatory contact. However, the response of the peripheral and central gustatory systems to these substances remains poorly explored. We measured the effect induced by pheromonal cuticular mixtures on (*i*) the electrophysiological response of peripheral gustatory receptor neurons, (*ii*) the calcium variation in brain centers receiving these gustatory inputs and (*iii*) the behavioral reaction induced in control males and in mutant *desat1* males, which show abnormal pheromone production and perception. While male and female pheromones induced inhibitory-like effects on taste receptor neurons, the contact of male pheromones on male fore-tarsi elicits a long-lasting response of higher intensity in the dedicated gustatory brain center. We found that the behavior of control males was more strongly inhibited by male pheromones than by female pheromones, but this difference disappeared in anosmic males. Mutant *desat1* males showed an increased sensitivity of their peripheral gustatory neurons to contact pheromones and a behavioral incapacity to discriminate sex pheromones. Together our data indicate that cuticular hydrocarbons induce long-lasting inhibitory effects on the relevant taste pathway which may interact with the olfactory pathway to modulate pheromonal perception.

## Introduction

Courtship behavior is regulated by multimodal sensory signals including vision, audition, olfaction and gustation [1]–[3]. Many insects have developed an acute chemical communication system to detect and orient to their mate at a far distance [4]. In some species, including many *Drosophila* species, sex pheromones are also perceived at a short distance or by physical contact to regulate courtship and mating behavior between potential sex-partners [5], [6].

In *Drosophila melanogaster*, both olfactory and gustatory sex pheromones are used for mate recognition and choice [7]. The only known olfactory pheromone is *cis*-vaccenyl-acetate (cVA), a compound produced by males and transferred to females during copulation and subsequently deposited on the food during egg laying [8]. At long distance and in synergy with volatile food molecules, cVA induces aggregation behavior [9]. This allows flies to meet and court on the same food source. At a short distance, cVA tends to inhibit male courtship and to stimulate female sexual receptivity [10], [11]. While cVA is only displayed during social (or sub-social) interactions [12], cuticular hydrocarbons (CHs) which cover the fly cuticle are thought to be received by contact or at a short distance when the flies beat their wings [13]–[16]. Among the 59 CHs that flies produce with very different abundance (between 1 and 1000 ng/fly; [12]), some of them show qualitative and quantitative variations between the sexes. Both sexes produce (*Z*)-7-tricosene (7-T), but this CH is very abundant in males. Differently, only females produce (*Z,Z*)-7,11 heptacosadiene (7,11-HD). 7-T and 7,11-HD respectively tend to inhibit or stimulate male sexual ardor [16], [17]. Moreover, the level of these CHs can vary between wild-type flies of different geographic origins: Tai females mostly produce (*Z,Z*)-5,9 heptacosadiene (an isomer of 7,11-HD) whereas Tai males produce large amounts of (*Z*)-7-pentacosene and low 7-T [6], [18]. Flies of the mutant-induced *desat1* strain are defective for the production of desaturated CHs and show low levels of both pheromones. Moreover, *desat1* males are defective in the perception of these pheromones [19].

The taste neurons involved in the pheromonal perception remains poorly known. Among peripheral appendages potentially involved in taste, the labellum harbors three types of taste sensilla: short, long and intermediate (s-, l- and i-types; [20]). s- and l-type sensilla contain four gustatory receptor neurons (GRNs) responding to sugar (S), water (W), low concentration of salt (L1) and aversive compounds (L2) whereas i-type sensillum contains two GRNs including one L2 neuron. We previously showed that 7-T is detected by L2 neurons of s- and i-type sensilla of *Gr66a*-expressing neurons harbored in the labellum [16]. The same neuron also responds to food compounds inducing repulsive behavior [21], [22]. Some *Gr66a*-expressing neurons of the tarsi also harbor either Gr32a or Gr33a taste receptors involved in the perception of an unknown pheromone inhibiting male courtship [23], [24]. *Gr66a*-expressing taste neurons project into a neural region of the sub-oesophageal ganglia (SOG) involved in the response to aversive substances whereas a distinct SOG area receive appetitive inputs (such as those induced by sugar) of *Gr5a*-expressing neurons [25]. It is not known whether GRNs responding to unknown female pheromone(s) stimulating male courtship also project to the latter SOG area [26], [27].

Given that the physiological response of the taste nervous system to the complete cuticular pheromonal bouquet remains unknown, our principal aim was to establish a link with the behavioral effects induced by such a bouquet on the male fly. Therefore, we measured the responses elicited by the pheromonal mixture on (*i*) gustatory receptor neurons, (*ii*) their projection in the central nervous system and (*iii*) the behavior of the fly. Our goal was to link these three integrative levels to better understand how information about CHs is processed along different levels of the taste neural pathway. We also measured the response in *desat1* mutant males defective for pheromonal discrimination [19] and we manipulated the olfactory system to assess whether it could interact with taste perception to modulate pheromonal perception.

## Results

### Electrophysiological response of labellar sensilla

**Figure 1:**
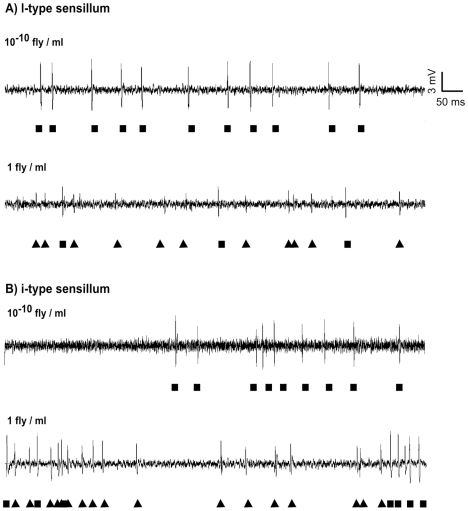
Responses of two labellum sensilla to CS male extract. Electrophysiological responses to male extract were recorded in l-type (A) and i-type (B) sensilla using the tip recording method. Spikes were classified into two classes (small, large) according to their amplitude and shape (small spikes correspond to the filled triangles and large spikes to the filled squares). The concentration of extracts is indicated above each trace. At one fly/ml concentration, each ml of the CS male extract contained about 1.2 ug cuticular hydrocarbon (including 0.5 ug 7-T). A dual scale for time and amplitude is shown on the top right.

**Figure 2:**
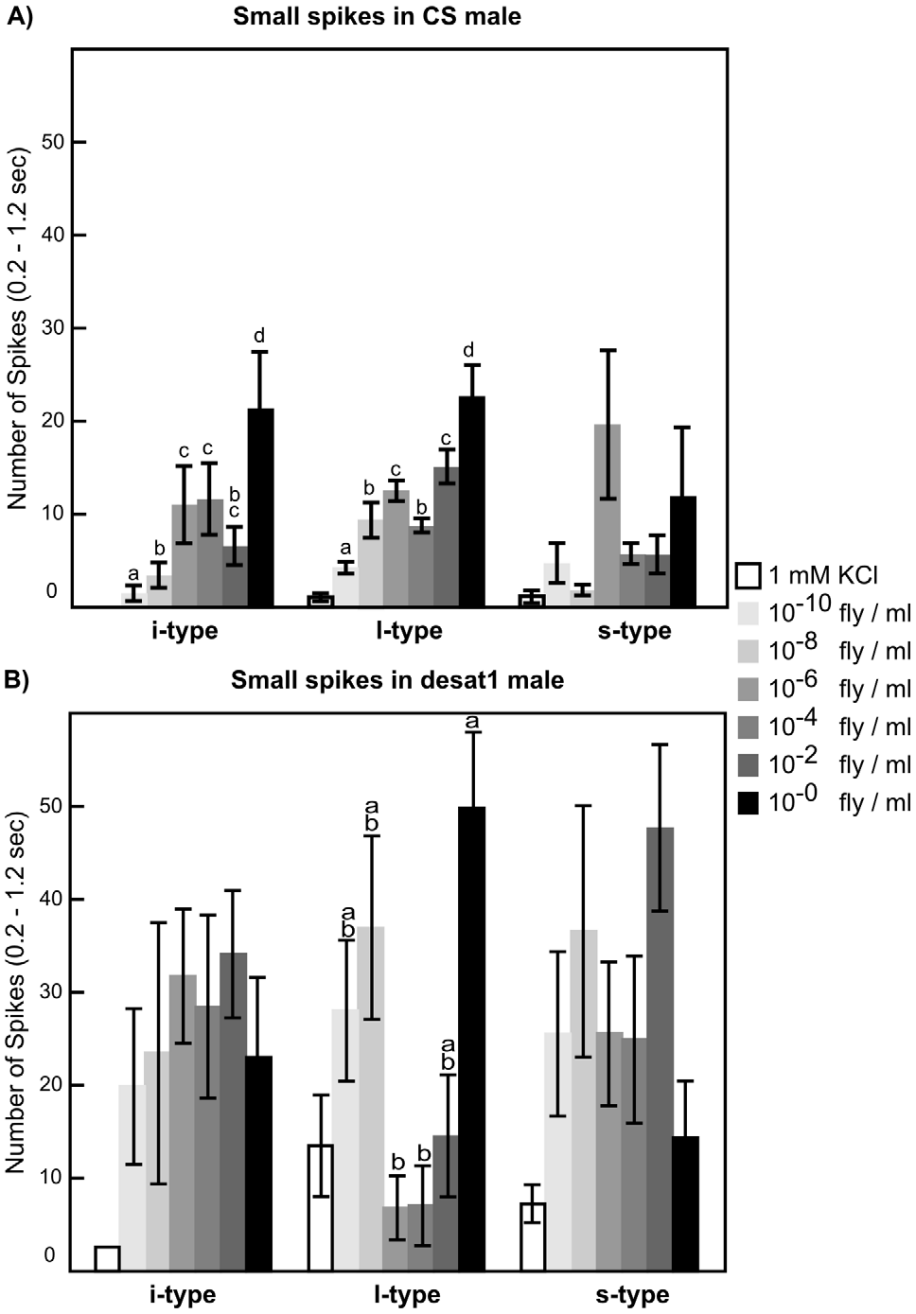
Dose-dependent response of three labellum sensilla to CS male extracts. Responses in CS males (A) and in *desat1* males (B) were measured to control solution (1mMKCl and 5% ethanol empty bars) and variable concentrations of CS male extracts (10^−10^ to 1fly/ml; light to dark filled bars) diluted in the control solution. Histogram bars represent the number of spikes during one sec (from 0.2 sec to 1.2 sec after stimulation). These data were compared within each sensilla-type with a Kruskal-Wallis test for CS males: i-type: p = 0.003, l-type: p = 0.002; N = 5–34; For *desat1* males: l-type: p = 0.0004; N = 9–34). The different letters (a–d) shown above the bars indicate the statistical differences.

We used the tip recording method to record electrophysiological responses of labellar sensilla in both wild-type CS and mutant *desat1* males to whole cuticular hydrocarbon (CH) extracts of CS males. These extracts elicited dose-dependent firing activity in i-, s- and l-type sensilla, characterized by spikes of two amplitudes (large and small; shown respectively as squares and triangles on Figure 1). In CS males, the number of small amplitude spikes increased with the extract concentration in i- and in l-type sensilla (Figures 1 and 2A) while the number of large amplitude spikes remained fairly constant (data not shown). Since 7-T stimulates L2 cells in i-type sensilla [16], we presume that these small amplitude spikes originate from L2 cells which responded in a dose-dependent way to the CH blend in these sensilla. However, we did not find any dose-dependent response to CS male extract in s-type sensilla which nevertheless responded to this blend.

Unexpectedly, in *desat1* males, the L2 neurons of the 3 types of sensilla were very sensitive to the CH blend (Figure 2B). This higher sensitivity was also associated with a higher inter-individual variability than in CS males. In addition, neurons from l-type sensilla showed a U-shaped response to a range of dilutions of the CH blend, with maximal responses at the lowest dilutions (10^−10^ to 10^−8^ fly/ml) and at the highest one (1 fly/ml). Such a change of peripheral taste response in *desat1* males may explain their defective pheromonal perception [19].

### Electrophysiological response of tarsal sensilla

**Figure 3:**
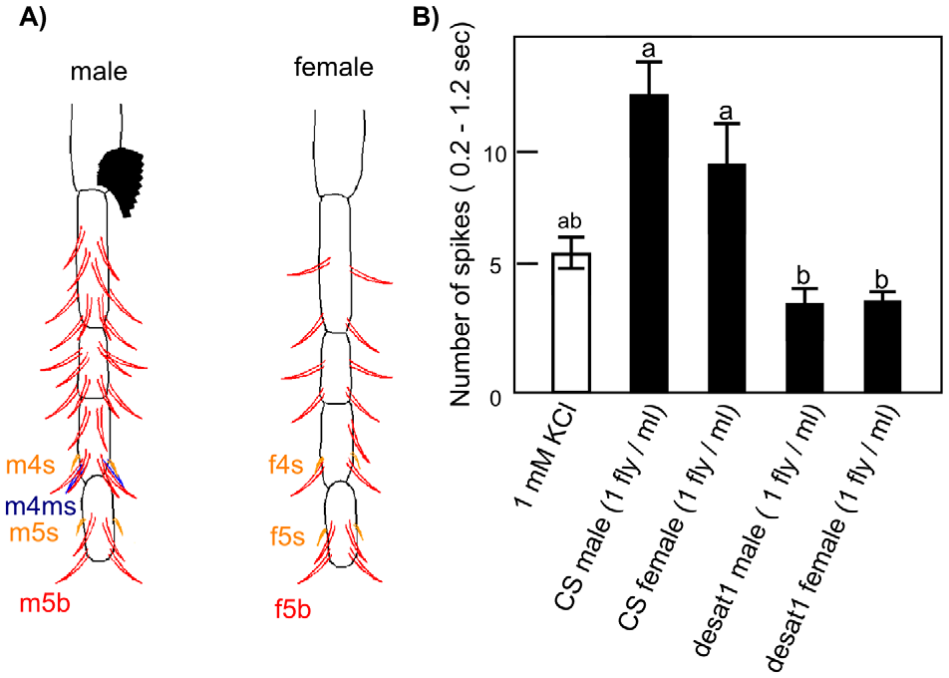
Response of a male tarsus sensillum to various extracts. Comparison of the fore-tarsi taste sensilla distribution between the sexes and location of the m4ms male-specific sensilla (A). The response of m4ms was measured to extracts of CS male and female, *desat1* male and female (B). All extract solutions included 5% ethanol and 1 mM KCl. Responses to CS male and *desat1* male extracts (but not between CS male and female extracts) significantly differed (F-tests with the correction of Bonferroni; N = 9–30). The different letters (a,b) shown above the bars indicate the statistical differences.

We also measured electrophysiological responses of taste sensilla located on the tarsi. We focused our study on one male-specific sensilla (m4ms; Figure 3 A) whose responses to other compounds are already known [28]. This sensillum clearly responded to the CHs extracts from two sexes of the two genotypes (CS male and female, *desat1* male and female) which strongly differ in their principal CHs (Table 1; [16], [19]; see Material and methods). The stimulation of the m4ms sensilla with a control solution (containing 1 mM KCl in 5% ethanol) elicited both large and small amplitude spikes. Male and female CS extracts (diluted in the control solution) elicited a relatively high number of small amplitude spikes. This response was significantly lower with extracts from *desat1* male and female flies. Although the responses to CS flies extracts were clearly higher than to the control solution, they showed no significant difference.

**Table 1:** Production of the principal cuticular hydrocarbons in CS and desat1 flies.

Genotype	7-T	23Lin	7-P	25Lin	7,11-HD	27Lin	7,11-ND	∑CHs
**Cs male**	467±58	168±22	170±17	29±5	0	11±2	0	1219±63
**Cs female**	53±9	102±13	89±10	120±14	447±45	27±6	201±36	1814±45
**desat1 male**	108±12	1262±99	32±3	337±17	0	101±7	0	2491±67
**desat1 female**	16±4	355±26	48±4	670±39	25±3	296±17	17±2	2088±89

Data represent the mean (± s.e.m.) in ng for the different compounds in single 4-days old flies. For the sake of clarity, we only show the most abundant hydrocarbons which are: 7-tricosene (7-T), n-tricosane (23Lin), 7-pentacosene (7-P), n-pentacosane (25Lin), 7,11-heptacosadiene (7,11-HD), n-heptacosane (23Lin), 7,11-nonacosadiene (7,11-ND). We also show the sum of all hydrocarbons (∑CHs). Note that if absolute quantities can change with time, their proportion remain very constant (7). N = 25 for all genotypes.

### Calcium activity detected in the central nervous system

**Figure 4:**
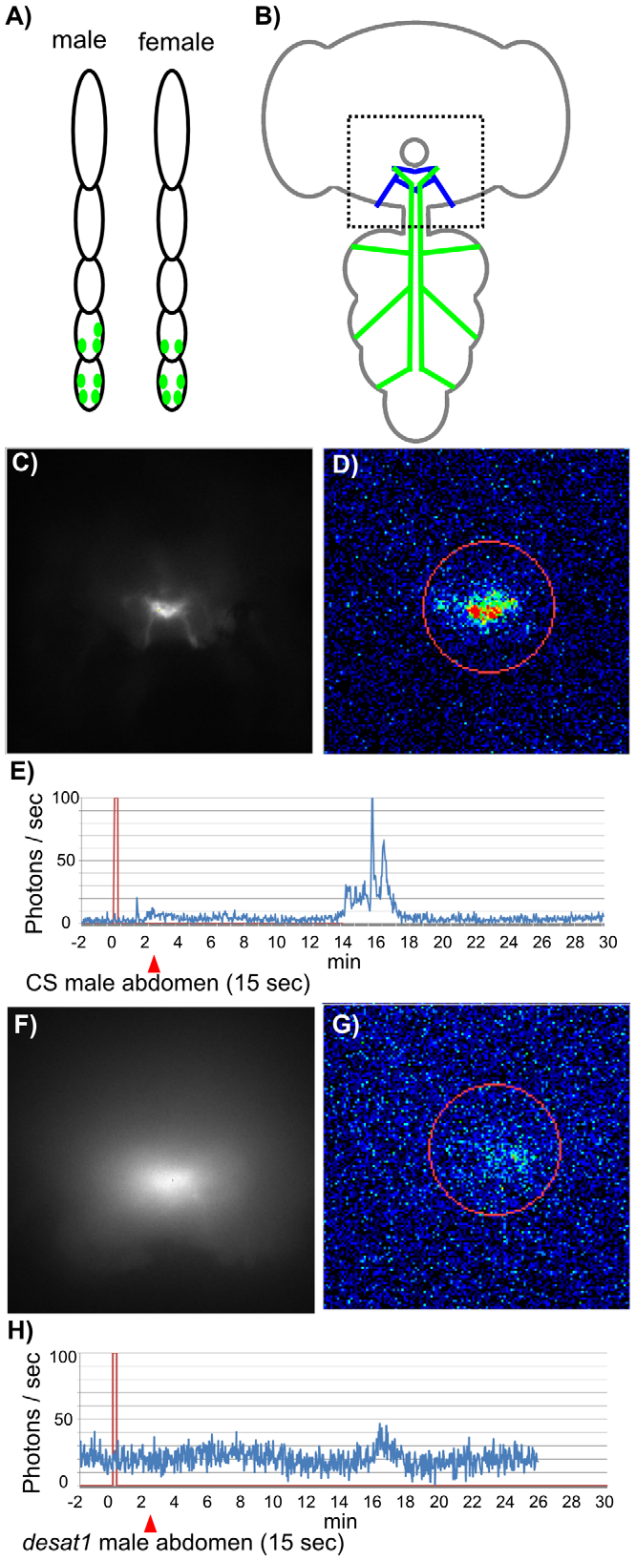
Ca^2+^-response in targeted neurons of the sub-eosophageal ganglia after tarsal stimulation. The top diagrams show the expression pattern of *Gr66a*-expressing neurons in male and female fore-tarsi (green, A) and their projection pattern in the CNS (B). In B, the green and blue lines respectively represent the axons from the legs and from the labellum expressing GFP-aequorine, the area delimitated with a broken line box represents the brain region highlighted in our imaging study and the circle symbolizes the oesophagus. The response of *Gr66a-Gal4;UAS-GA* males was measured after the stimulation with the cuticle of a CS male (C–E) and a *desat1* male (F–H). C and F represent the fluorescent images of axon terminal of GRNs within the SOG, taken before the Ca^2+^-activity recording, and used as reference image. D and G represent the bioluminescent image of Ca^2+^-activity induced by the stimulus (60 sec accumulation time). E and H represent the profile of the whole Ca^2+^-response following the stimulus (red triangle). The red circle (in D, G) represents the region of interest used to quantify the Ca^2+^-activity (number of emitted photons/sec presented in E and H). The bioluminescent activity (indicated in photons/sec; E, H) is shown as a function of time (in min) after a 15 sec stimulation (symbolized by the red bar). In control CS males, a weak signal was elicited after 2–3 min (E) in the SOG projection area of fore-tarsi *Gr66a*-targeted neurons. Moreover, after 14–16 min, a stronger signal, which lasted for about 4 min, was recorded. This delayed Ca^2+^-response reached a higher amplitude after the stimulation with CS male cuticle (peak at 100 photons/sec) than with *desat1* male cuticle (peak at 45 photons/sec), which also lasted for a shorter period (about 1 min).

Since most labellar and tarsal gustatory receptor neurons (GRNs) conveying inhibitory inputs project to a specific area of the sub-oesophageal ganglia (SOG; Figure 4A,B), we targeted this region with a bioluminescent Ca^2+^-reporter *GFP-Aequorin* (*GA*; [29]). This allowed us to monitor the response of the central nervous system to CHs. More specifically, the variation of *GA* was targeted by *Gr66a*-expressing GRNs in the SOG and monitored after the application on the taste sensilla of tester males of whole pheromonal stimuli of different genotypes. We stimulated taste sensilla either with (*i*) with a tip electrod filled with the whole CH extract or (*ii*) with a piece of abdominal cuticle that was gently rubbed on sensilla. We kept the latter approach which has the advantage of being closer to the natural stimulation.

The stimulation of the CS male tarsus with CS male abdominal cuticle induced a low-amplitude response (above the background noise level) in about 2/3 of males (N = 17/27; Figure 4C–E). These low responses had a relatively long duration (between 3 and 10 min). Moreover, one or two signals of higher amplitude were detected 6 to 30 min after the application of the stimulus, in one third of the flies (9/27). In several cases, the stimulation with the cuticle of *desat1* male (6/12; Figure 4F–H) or of CS female (5/14; not shown) induced long lasting signals of very low amplitude. Larger size-amplitude signals were also induced by the cuticle of these two genotypes (in respectively 4/12 and 2/14 cases). These signals appeared with a similarly delayed timing as that induced by the CS male cuticle. However, the response induced by the CS male cuticle seemed to be stronger and to last longer than that induced by *desat1* male cuticle.

We also measured the response of labellar sensilla both to non-pheromonal and pheromonal stimulation (Figure S1). The stimulations induced by (*i*) a piece of paper filter impregnated with quinine or (*ii*) a tip electrod filled with a CS male extract both elicited delayed physiological changes similar to those induced by CS male abdominal cuticles. This indicates that the two changes induced by fly cuticles on the tarsa were specifically caused by cuticular pheromones. We can also rule out the effect of a mechanical stimulation since *GA* was targeted in *Gr66a*-Gal4 taste specific neurons.

In summary, the contact of a fly cuticle on the fore tarsi generally induced two successive Ca^2+^-bioluminescent responses in the inhibitory area of the SOG: a long-lasting response (∼10 min) with a low amplitude which was followed by a shorter response (∼4 min) of a higher amplitude.

In contrast, no response was detected in response to pheromonal stimulation in flies with *Gr5a*-expressing neurons targeting GA in SOG (data not shown).

### Behavioral response and suppression of the proboscis extension reflex

**Figure 5:**
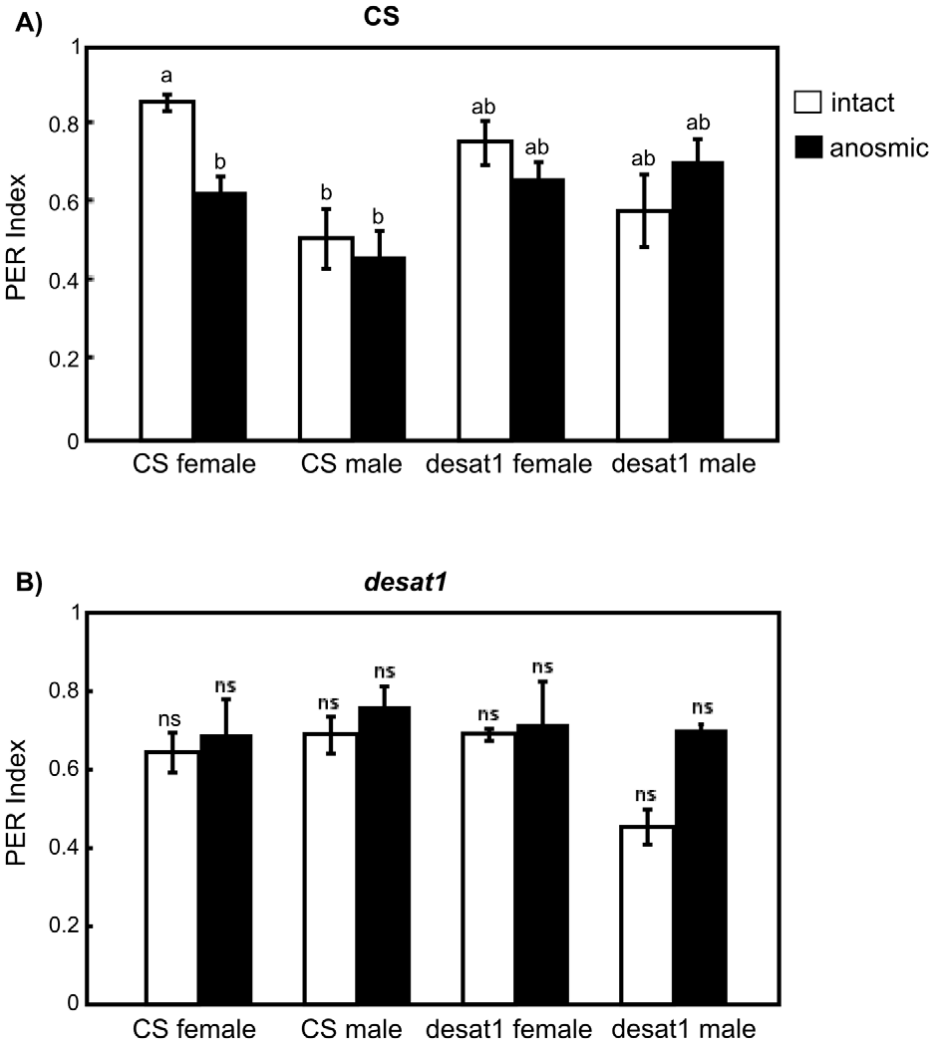
Behavioral responses of CS and *desat1* males to cuticle of various flies. Histograms indicate the average (± sem) of individual proboscis extension reflex (PER) indexes in response to the abdominal cuticle of CS female, CS male, *desat1* female and *desat1* male. Flies who extended their proboscis and opened their labellum were counted as PER positive flies. The response was measured in CS males (A) and in *desat1* males (B). These males were either intact (empty bars) or anosmic (filled bars; with antennae and maxillary palps surgically removed). The PER index is shown relatively to the positive response of individual flies to unilateral stimulation by sucrose ( = 100%). The bilateral stimulation (sucrose + fly cuticle) allows to estimate the inhibiting effect induced by the fly cuticle on the PER. (A) Intact but not anosmic CS males showed a different response to CS female and CS male cuticle (p = 0.014; one-way ANOVA, Holm test). N = 46–116. (B) Intact and anosmic *desat1* males showed no significant difference (p = 0.24). N = 56–96. The different letters (a, b) shown above the bars indicate the statistical differences.

To assess the inhibitory effects of CHs on behavior, we measured their ability to suppress proboscis extension reflex (PER) in male flies initially stimulated with sucrose. The PER is a useful test to measure the fly behavior in response to sex pheromone (or to food molecules) applied on the male fore-tarsus [16]. We stimulated one fore-tarsus of a male fly with a sucrose solution (to elicit PER) and we immediately touched the contralateral fore-tarsus with an abdominal cuticle. To estimate the inhibitory effect induced by CHs (carried on the cuticle), we calculated the difference observed between the number of PER induced (*i*) on flies unilaterally stimulated by sucrose and (*ii*) on flies bilaterally stimulated by sucrose and CHs (Figure 5; a PER index equal to 1 represents 100% responses indicating no suppression).

We compared the response of CS and *desat1* males (Figure 5) either intact (empty bars) or surgically deprived of their olfactory organs (anosmic; filled bars). This allowed us to assess the role of olfactory cues on PER on wild-type and mutant flies. In other words, the purpose of this experiment consisted to study the effects of gustatory and olfactory stimuli from the cuticle since no other stimulus source was presented. As in the previous section, we stimulated these males with a piece of abdominal cuticle of CS and *desat1* flies of both sexes. In intact CS males, the CS male cuticle induced a stronger PER repression than that of CS females whereas the cuticle of *desat1* flies induced a intermediate effect (Figure 5A). More specifically, about 10 and 50% PER positive responses were suppressed respectively by CS female and male cuticles whereas *desat1* female and male cuticles respectively suppressed 25 and 40% PER. The ablation of olfactory organs in CS males only significantly affected their response to the CS female cuticle: anosmic tester CS males showed 25% less PER than intact siblings. Since olfactory deprivation induced no significant variation in response to the cuticle of the three other genotypes, all stimuli induced a similar PER index.

In contrast, *desat1* tester males showed no significant difference for PER index whatever the cuticle used or the male treatment (intact, anosmic; Figure 5B). The bilateral tarsal contact with any fly cuticle repressed PER in about 30–40% of the cases (50% with *desat1* male cuticle in intact males).

## Discussion

Our data provide new insights on the link between the peripheral and the central physiological responses of the nervous system to sex pheromone in relation with the behavioral response of *Drosophila* males. Moreover, the comparison of peripheral taste responses between control and *desat1* males may explain the altered pheromonal discrimination of mutant males.

### Peripheral activity of taste neurons

L2 GRNs respond to inhibitory tastants (bitter substances, 7-T and high salt) in s- and i-type sensilla on the proboscis (in l-type sensilla , their ligands are not known yet). L1, S and W GRNs respond to phagostimulatory tastants and are present in l- and s-type sensilla whereas i-type sensilla are mostly devoid of L1 and W GRNs. Since most L2 GRNs express Gr66a, while most S GRNs express Gr5a, these neurons were shown to project in different areas of the SOG [25]. Our data indicate that L2 neurons of i-type and l-type labellar taste sensilla responded to the whole CS male CH extract (in the range of 10^−10^ to 1 fly/ml), but not in s-type sensilla. This result contrasts with the effect induced by the principal CH of CS males, (*Z*)-7-tricosene (7-T; [16]) which elicited a clear electrophysiological response in i-type and s-type sensilla — but not in l-type sensilla. L2 neurons of i-type sensilla might be specifically tuned to 7-T since they showed very similar dose-response curves to 7-T and to CS male extract ([16]; this study). L2 neurons from s-type sensilla responded to 7-T but not to the CS extract over the whole concentration range used here; we presume that these neurons were inhibited by some compounds of the blend. Lastly, L2 neurons from l-type sensilla, which do not respond to 7-T [16], showed a dose-dependent response to CS male extract; this suggests that they respond to other CHs than 7-T. Based on these observations, we hypothesize that the different types of labellar sensilla have a different spectrum of response to contact pheromones.

If *Gr32a* plays a significant role in the modulation of male courtship behavior [23], [24], this receptor molecule is not expressed in the l-type sensilla of the labellum [20], [30] which responded to the CS male cuticular extract. This indicates that other yet unknown receptor gene(s) expressed in the labellum is (are) involved in the response to cuticular pheromones. Based on the varied pattern of expression — and of co-expression — of several Grs in L2 cell from labellar sensilla [20], [30], we hypothesize that the interindividual variability of their responses to CHs extract is related to the non-uniform distribution of sex pheromone receptor genes in these sensilla.

Beside 7-T, many other male CHs potentially involved in the sexual interaction could have a pheromonal effect [12], [13]. Some of these compounds, not yet characterized, and inhibiting male courtship are detected by the GR32a and GR33a receptor molecules which are located on tarsal sensilla [23], [24]. The fact that these Grs are also found on *Gr66a*-expressing neurons — involved in the detection of 7-T and bitter substances — suggest that the corresponding GRNs co-express different Grs involved in the reception of aversive substances. Moreover, since Gr32a is also involved in the perception of a yet unknown female pheromone stimulating a specific aspect of male courtship [27], other Gr(s) could interact with diverse compounds of the pheromonal bouquet to modulate behavior.

Stimulated L2 neurons of i- and s-type sensilla showed a higher spiking frequency in *desat1* mutant males than in CS males. However, the lack of dose-dependent response in *desat1* taste neurons suggest that they are more sensitive than CS male neurons to CS male extract. This may explain the reduced ability of *desat1* to behaviorally discriminate sex pheromones (Figure 5B; [19], [31]).

### Integrating pheromonal signals in the brain

We also found coherent physiological changes between the peripheral and central taste nervous system following the stimulation of tarsal sensilla. Our data show that the m4ms male-specific taste sensillum more intensively responded to the extract of CS flies than that of *desat1* flies. The projection region of *Gr66a*-expressing neurons in the SOG of CS males showed two physiological changes (Ca^2+^-response) induced by the stimulation with cuticular pheromones of CS males: (*i*) a rapid response that may correspond to the stimulation of the GR by its ligand, and (*ii*) a delayed response after about 15 min. The cuticle of *desat1* male (and CS females) also induced a rapid and a delayed response, both of much weaker intensity, compared to those induced by CS males.

These findings not only raise the potential ability of peripheral tarsal taste neurons to discriminate contact pheromones but also suggest that these substances can have strong long-lasting behavioral effects. The changes shown by labellar *Gr66a*-Gal4 neurons in response to quinine and Cs male extracts suggest that the long lasting effect is related to the intrinsic properties of *Gr66a*-expressing neurons conveying inhibiting influx ([16], [21], [22]; Figure S1). This long lasting effect was revealed due to the particular characteristics of the *GA* bioluminescent marker which allows continuous recording over a long range period (from several minutes up to hours). This contrast with other fluorescent Ca^2+^-activity probes [25], [32], [33], which are generally used to detect [Ca^2+^] modifications over much shorter time periods (few seconds) after the stimulus application. Moreover, the *GA* bioluminescent probe does not requires light excitation and is not altered by undesirable side effects such as those induced by auto-fluorescence, photobleaching and phototoxicity [29], [34]. Interestingly, the delayed response observed here resembles that induced by acetylcholine (and nicotine) and exclusively observed in the mushroom-bodies (MBs) lobes [29]. This phenomenon was shown to depend on changes in the contraction of Ca^2+^ from intracellular stores, such as endoplasmic reticulum. However, we do not know whether the delayed response observed here also depends on the intracellular Ca^2+^-stores in the GRNs. Moreover, the delayed association of a strong physiological change elicited by some inhibitory contact pheromones could reinforce, similarly to spaced repeated stimuli, an associative memory process inducing aversive courtship conditioning behavior [35]. Interestingly, courtship conditioning memory related, or not, to cuticular pheromones is observed after few minutes [36], [37] and can last several days [38], [39]. It may be enhanced if the exposure to male cuticular pheromones occurs during a critical period corresponding to sexual immaturity [40].

### Behavioral effect of pheromones in control and *desat1* males

Since males without olfactory organs showed an increased PER suppression, this suggests that the female pheromone(s) detected by intact male olfactory organs have a stimulating effect that reduce the inhibitory effect of CHs perceived by taste. These hypothetical volatile (or semi-volatile) pheromones should only be found on the cuticle of control CS females since no other genotype induced a significant difference in anosmic tester CS males. Moreover, these olfactory female pheromones may not be perceived by *desat1* males since olfactory deprivation did not affect their PER to CS females. If the precise biological mechanism by which the *desat1* mutation alters pheromonal perception remains unknown, our current data reveal that this gene is expressed in large basiconic and trichoid olfactory sensilla located on the third antennal segment and projecting to antennal lobes glomeruli previously implicated in pheromonal perception (F. Bousquet, JF. Ferveur., unpublished data). The alternative hypothesis suggesting that *desat1* physiological and behavioral defects are the consequences of adaptive changes resulting of different self-exposure of the two males to their diverging cuticular profiles can be ruled out since the alteration of the two pheromonal phenotypes (production/discrimination) were genetically dissociated [31].

In summary, our data indicate that cuticular pheromones of control males induce both a rapid and a long-lasting effect in the brain of control males that could explain their durable aversive effect. Moreover, some female pheromones, perceived both by taste and olfactory pathway, may serve to modulate male perception and sexual response. The inability of *desat1* males to detect sex pheromones could result both of the increased excitability of their taste sensilla and the impairment of olfactory organs.

## Materials and Methods

### Fly culture and strains

All *Drosophila melanogaster* stocks were kept on yeast/cornmeal/agar medium at 24±0.5°C on a 12∶12 hr light/dark cycle. We used Canton-S strain (CS) as wild type strain and *desat1* mutant strain in electrophysiological recordings, calcium imaging study and behavioral tests. *Gr5a-Gal4*, *Gr66a-Gal4* and *UAS*-*GFP*-aequorin [29] have been used in trans-heterozygous flies for Ca^2+^- imaging study.

### Cuticular hydrocarbons extraction

We followed a standard procedure [41]. Briefly, to obtain total cuticular extracts, individual four-day old virgin flies were washed into hexane (1fly/30 µl) during five minutes and immediately removed. After the complete evaporation of the solvent, the extract was dissolved into 5% ethanol.

### Electrophysiology

We used a tip recording method to record electrophysiological responses from taste sensilla [42]. A glass capillary tube filled with Drosophila Ringer solution mixed with the cuticular hydrocarbon extract was inserted into fly abdomen as an electrical ground. Glass capillaries with a tip diameter of 15 to 20 µm were used as recording/stimulating electrodes. The electrode was connected to a TasteProbe amplifier (SYNTECH, Hilversum, The Netherlands). The signal was further amplified with a CyberAmp 380 (Axon Instruments, Union City, CA; gain ×1,000; 8^th^ order Bessel pass-band filter, 1–2,800 Hz). Each taste sensillum was briefly capped with the stimulus electrode (during 2 s) in order to establish an electrical contact and to record the response of the taste neurons to the stimulus. Electrical signals were sampled by Digidata1440A (Molecular Devices, Chicago, IL) and analyzed using pCLAMP and ClampFit software (Molecular Devices, Chicago, IL). We analyzed data between 0.2 sec to 1.2 sec after the stimulation.

We recorded the responses from a subset of labellar sensilla: from i-type (i5-i8), l-type (l3-l7) and s-type (s2 and s6), and also from a male specific sensillum on the 4th segment of the male fore-tarsus (m4ms; Figure 3). Spikes were classified according to both their amplitude and shape.

### *In vivo* calcium imaging

To record physiological activity elicited by the stimulation within the axon terminals of GRNs of the SOG, we used *in-vivo* bioluminescent Ca^2+^-reporter *GFP-Aequorin* (*GA*). GA was expressed in *Gr66a*-expressing neurons by crossing *UAS-GA* transgenic flies with *Gr66a-Gal4* transgenic flies. Similar experiments were carried out with *Gr5a-Gal4*. Three to five days-old transgenic males carrying both *Gr66a-Gal4* (or *Gr5a-Gal4*) and *UAS*-*GA* were fixed on plastic coverslip by their neck. The proboscis, antenna and maxillary palps were removed and a small hole was made in the cuticle covering the SOG. Drosophila's Ringer solution with the cofactor coelenterazine (1,5 µM) was applied on the SOG and incubated into a dark box (with a saturated vapor, 24.5°C) during 5 hrs [29].

The fore-tarsi and labella of transgenic *Gr66a-Gal4;UAS-GA* flies were stimulated using whole cuticular pheromone extracts and abdominal cuticles of single flies. To stimulate fore-tarsi with the fly abdominal cuticle, we used a motorized manipulator. The fly abdomen was moved laterally (20 micrometers) during 15 sec to stimulate several sensilla on the tarsus. A similar approach has been recently used to test the effect of female pheromone on male courtship [43]. Tested flies were mounted under microscopy 15 to 30 min before stimulation. No spontaneous response was recorded before stimulation. The bioluminescent response of *GA*-expressing neurons was observed under the microscopy. Data were acquired and analyzed as previously described [29]. To measure the response of the *Gr66a*-Gal4 neurons of the labellum to pheromonal and non-pheromonal, we respectively used the tip electrod stimulation and small pieces of filter papers impregnated with quinine.

### PER tests

The proboscis extension reflex (PER) test was performed as described by Kimura et al. [44]. We starved three to five days old flies with water-saturated paper during 20 hrs at 25°C. To make flies anosmic, we surgically removed their antenna and maxillary palps one day before starvation. Flies to be tested were fixed on the glass slide and kept in a chamber saturated with humidity. Flies were also given water before each test. We scored the response of flies under a binocular microscope (Leica MZ8). First, we measured their responses following stimulation of one fore-tarsus with a 10 mM sucrose solution. Then, we measured their response following the bilateral stimulation of both fore tarsi: sucrose on one side and the pheromonal bouquet (abdominal fly cuticle) on the contralateral side. The repression of PER index was determined as the ratio of flies responding in the bilateral stimulation compared to those responding to sucrose alone. Note that although sugar stimuli are not rewarded in our test, the same flies extended their proboscis by after a second stimulation with sugar. Moreover, to avoid any habituation effect, fore-legs were washed with water between each test.

## Supporting Information

Figure S1 **Ca^2+^-response in targeted neurons of the sub-eosophageal ganglia after labellum stimulation with quinine (50 mM; A–C) and with a CS male extract (1fly/ml; D–F).** For further explanation, see the legend of Figure 4.
(TIF)

## References

[1] Hall JC (1994). The mating of a fly.. Science.

[2] Greenspan RJ, Ferveur JF (2000). Courtship in *Drosophila*.. Annu Rev Genet.

[3] Krstic D, Boll W, Noll M (2009). Sensory integration regulating male courtship behavior in *Drosophila*.. PLoS ONE.

[4] Howard RW, Blomquist GJ (2005). Ecological, behavioral, and biochemical aspects of insect hydrocarbons.. Annu Rev Entomol.

[5] Antony C, Jallon JM (1982). The chemical basis for sex recognition in *Drosophila melanogaster*.. J Insect Physiol.

[6] Jallon JM (1984). A few chemical words exchanged by *Drosophila* during courtship and mating.. Behav Genet.

[7] Ferveur JF (2005). Cuticular hydrocarbons: their evolution and roles in *Drosophila* pheromonal communication.. Behav Genet.

[8] Zawistowski S, Richmond RC (1986). Inhibition of courtship and mating of *Drosophila melanogaster* by the male-produced lipid, *cis*-vaccenyl acetate.. J Insect Physiol.

[9] Bartelt RJ, Schaner AM, Jackson LL (1985). cis-Vaccenyl acetate as an aggregation pheromone in*Drosophila melanogaster*.. J Chem Ecol.

[10] Kurtovic A, Widmer A, Dickson BJ (2007). A single class of olfactory neurons mediates behavioural responses to a *Drosophila* sex pheromone.. Nature.

[11] van der Goes van Naters W, Carlson JR (2007). Receptors and neurons for fly odors in *Drosophila*.. Curr Biol.

[12] Everaerts C, Farine JP, Cobb M, Ferveur JF (2010). *Drosophila* cuticular hydrocarbons revisited: mating status alters cuticular profiles.. PLoS ONE.

[13] Ferveur JF, Sureau G (1996). Simultaneous influence on male courtship of stimulatory and inhibitory pheromones produced by live sex-mosaic *Drosophila melanogaster*.. Proc Biol Sci.

[14] Rybak F, Sureau G, Aubin T (2002). Functional coupling of acoustic and chemical signals in the courtship behaviour of the male *Drosophila melanogaster*.. Proc R Soc Lond B.

[15] Grillet M, Dartevelle L, Ferveur JF (2006). A *Drosophila* male pheromone affects female sexual receptivity.. Proc Biol Sci.

[16] Lacaille F, Hiroi M, Twele R, Inoshita T, Umemoto D (2007). An inhibitory sex pheromone tastes bitter for *Drosophila* males.. PLoS ONE.

[17] Antony C, Davis TL, Carlson DA, Pechine JM, Jallon JM (1985). Compared behavioral responses of male *Drosophila melanogaster* (Canton-S) to natural and synthetic aphrodisiacs.. J Chem Ecol.

[18] Jallon JM, Pechine JM (1989). A novel chemical race of *Drosophila melanogaster* in Africa.. Comptes Rendus de l'Academie des Sciences Serie II.

[19] Marcillac F, Grosjean Y, Ferveur JF (2005). A single mutation alters production and discrimination of *Drosophila* sex pheromones.. Proc Biol Sci.

[20] Hiroi M, Marion-Poll F, Tanimura T (2002). Differentiated response to sugars among labellar chemosensilla in *Drosophila*.. Zool Sci.

[21] Meunier N, Marion-Poll F, Rospars JP, Tanimura T (2003). Peripheral coding of bitter taste in *Drosophila*.. J Neurobiol.

[22] Al-Anzi B, Tracey WD, Benzer S (2006). Response of *Drosophila* to wasabi is mediated by painless, the fly homolog of mammalian TRPA1/ANKTM1.. Curr Biol.

[23] Miyamoto T, Amrein H (2008). Suppression of male courtship by a *Drosophila* pheromone receptor.. Nat Neurosci.

[24] Moon SJ, Lee Y, Jiao Y, Montell C (2009). A *Drosophila* gustatory receptor essential for aversive taste and inhibiting male-to-male courtship.. Curr Biol.

[25] Marella S, Fischler W, Kong P, Asgarian S, Rueckert E (2006). Imaging taste responses in the fly brain reveals a functional map of taste category and behavior.. Neuron.

[26] Bray S, Amrein H (2003). A putative *Drosophila* pheromone receptor expressed in male-specific taste neurons is required for efficient courtship.. Neuron.

[27] Koganezawa M, Haba D, Matsuo T, Yamamoto D (2010). The shaping of male courtship posture by lateralized gustatory inputs to male-specific interneurons.. Curr Biol.

[28] Meunier N, Ferveur JF, Marion-Poll F (2000). Sex-specific non-pheromonal taste receptors in *Drosophila*.. Curr Biol.

[29] Martin JR, Rogers KL, Chagneau C, Brulet P (2007). *In vivo* bioluminescence imaging of Ca2+ signalling in the brain of *Drosophila*.. PLoS ONE.

[30] Weiss LA, Dahanukar A, Kwon JY, Banerjee D, Carlson JR (2011). The molecular and cellular basis of bitter taste in Drosophila.. Neuron.

[31] Houot B, Bousquet F, Ferveur JF (2010). The consequences of regulation of desat1 expression for pheromone emission and detection in *Drosophila melanogaster*.. Genetics.

[32] Reiff DF, Ihring A, Guerrero G, Isacoff EY, Joesch M (2005). *In vivo* performance of genetically encoded indicators of neural activity in flies.. J Neurosci.

[33] Miyawaki A (2005). Innovations in the imaging of brain functions using fluorescent proteins.. Neuron.

[34] Martin JR (2008). *In vivo* brain imaging: fluorescence or bioluminescence, which to choose ?. J Neurogenetics.

[35] Siegel RW, Hall JW (1979). Conditioned responses in courtship behavior of normal and mutant *Drosophila*.. Proc Natl Acad Sci USA.

[36] Griffith LC, Verselis LM, Aitken KM, Kyriacou CP, Danho W (1993). Inhibition of calcium/calmodulin-dependent protein kinase in *Drosophila* disrupts behavioral plasticity.. Neuron.

[37] Siwicki KK, Riccio P, Ladewski L, Marcillac F, Dartevelle L (2005). The role of cuticular pheromones in courtship conditioning of *Drosophila* males.. Learn Mem.

[38] McBride SM, Giuliani G, Choi C, Krause P, Correale D (1999). Mushroom body ablation impairs short-term memory and long-term memory of courtship conditioning in *Drosophila melanogaster*.. Neuron.

[39] Siwicki KK, Ladewski L (2003). Associative learning and memory in *Drosophila*: beyond olfactory conditioning.. Behav Processes.

[40] Svetec N, Cobb M, Ferveur JF (2005). Chemical stimuli induce courtship dominance in *Drosophila*.. Curr Biol.

[41] Ferveur JF (1991). Genetic-control of pheromones in *Drosophila simulans*. 1. Ngbo, a locus on the 2nd chromosome.. Genetics.

[42] Fujishiro N, Kijima H, Morita H (1984). Impulse frequency and action-potential amplitude in labellar chemosensory neurons of *Drosophila melanogaster*.. J Insect Physiol.

[43] Kohatsu S, Koganezawa M, Yamamoto D (2011). Female contact activates male-specific interneurons that trigger stereotypic courtship behavior in *Drosophila*.. Neuron.

[44] Kimura K, Shimozawa T, Tanimura T (1986). Isolation of *Drosophila* mutants with abnormal proboscis extension reflex.. J Exp Zool.

