# Supplementary figures and images for: Peripheral, Central and Behavioral Responses to the Cuticular Pheromone Bouquet in Drosophila melanogaster Males
Source: PLoS One. 2011 May 20;6(5):e19770. doi: 10.1371/journal.pone.0019770 (PMC3098836; doi:10.1371/journal.pone.0019770)

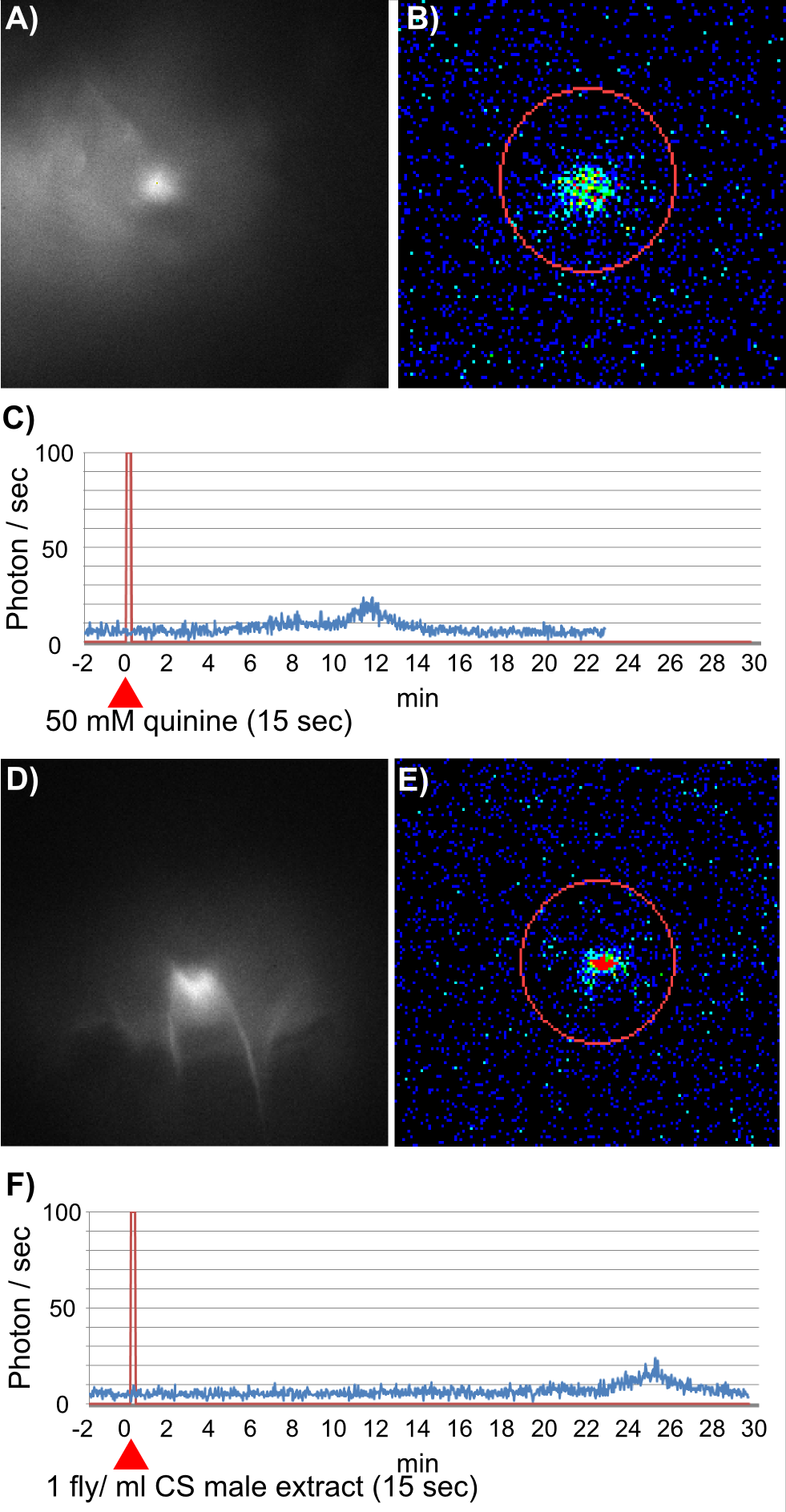

Supplement: Figure S1 — Ca2+-response in targeted neurons of the sub-eosophageal ganglia after labellum stimulation with quinine (50 mM; A–C) and with a CS male extract (1fly/ml; D–F). For further explanation, see the legend of Figure 4. (TIF) [file pone.0019770.s001.tif]
